# Gene therapy in glaucoma-3: Therapeutic approaches

**DOI:** 10.4103/0974-620X.71883

**Published:** 2010

**Authors:** Mohamed Abdel-Monem Soliman Mahdy

**Affiliations:** Department of Ophthalmology, Rustaq Hospital, Rustaq, Sultanate of Oman and Al-Hussein University Hospital, Faculty of Medicine, Al-Azhar University, Cairo, Egypt

**Keywords:** Gene therapy, genetic tests for glaucoma, glaucoma

## Abstract

Despite new and improving diagnostic and therapeutic options for glaucoma, blindness from glaucoma is increasing and glaucoma remains a major public health problem. The role of heredity in ocular disease including glaucoma is attracting greater attention as the knowledge and recent advances of Human Genome Project and the HapMap Project have made genetic analysis of many human disorders possible.

Glaucoma offers a variety of potential targets for gene therapy. All risk factors for glaucoma and their underlying causes are potentially susceptible to modulation by gene transfer. As genetic defects responsible for glaucoma are identified and the biochemical mechanisms underlying the disease are recognized, new methods of therapy can be developed. Genetic tests are indicated for treatment, diagnosis, prognosis, counseling, and research purposes; however, there is significant overlap among them. One of the important genetic tests for glaucoma is OcuGene. Therefore, it is of utmost importance for the glaucoma specialists to be familiar with and understand the basic molecular mechanisms, genes responsible for glaucoma, and the ways of genetic treatment.

Recently, several promising genetic therapeutic approaches had been investigated. Some are either used to stop apoptosis and halt further glaucomatous damage, wound healing modulating effect or long lasting intraocular pressure lowering effects than the conventional commercially available antiglaucoma medications.

**Method of Literature Search:**

The literature was searched on the Medline database using the PubMed interface. The key words for search were glaucoma, gene therapy, and genetic diagnosis of glaucoma.

## Genetic Approach

The identification of the molecular events responsible for glaucoma has been difficult because of a general lack of knowledge about the cellular and biochemical events necessary for the normal regulation of intraocular pressure (IOP) and retinal ganglion cell (RGC) function. Access to human diseased tissue is also difficult, and animal models have only recently been developed and characterized. The advantage of a genetic approach is that the responsible protein can be identified without access to diseased tissue. The identification of genes (and their protein products) that can cause or contribute to glaucoma will help define the underlying pathophysiology, as well as lead to the development of new DNA-based diagnostic tests and novel therapeutic approaches.[1]

The availability of predictive tests would provide a mechanism for early detection and treatment. Those individuals at risk who are identified early in the course of the disease and who begin therapy prior to significant damage to the optic nerve will have the best chance of maintaining useful vision.

## What are the Available Genetic Tests and its Indications?

In the broad sense, a genetic test is any clinical or laboratory maneuver that has the potential to increase or decrease the likelihood that a patient has an inherited disease. Thus, an abnormal electroretinogram in an asymptomatic 10-year-old child of a parent with autosomal-dominant retinitis pigmentosa is as much a genetic test as a molecular investigation of the rhodopsin genes.[2]

A knowledgeable clinician is arguably the single most important component of the genetic testing process. Indeed many physicians, laypeople, and regulatory agencies often have a much narrower view of a genetic test. They tend to see it as the performance of one or more laboratory techniques that result in a black-and-white answer about the presence or absence of a disease-causing mutation. At least four features of a genetic test are of interest to a clinician and patients as well; the cost, the turn-around time, the report, and the likelihood that the test will assist in the management of the patient. Many tests are available for inherited eye diseases such as glaucoma [Table 1].[2,3] These tests are available at different laboratories in many schools of medicines in USA, Canada, and at the National Institute of Health, USA.[2]

**Table 1:** Inherited Glaucomas with available genetic testing.[2]

*Test/Diagnosis*	*Inheritance Pattern*	*Gene*
Aniridia	AR	PAX6
Juvenile open-angle glaucoma	AD	MYOC
Primary congenital glaucoma	AR	CYP1B1
Primary open-angle glaucoma	AD	MYOC
Rieger syndrome	AR	FOXC1 and PITX2

Autosomal recessive = AR, Autosomal dominant= AD

The indications for genetic testing can be divided into five broad categories; treatment, diagnosis, prognosis, counseling, and research. However, there is significant overlap among them.[2] One of the important genetic tests for glaucoma is OcuGene.

## OcuGene

InSite Vision (Alameda, CA) recently released a diagnostic kit for primary open-angle glaucoma (OcuGene) based on the TIGR/MYOC mt1 variant in the promoter region of the gene.[4] OcuGene is the first commercialized genetic test that screens for the presence of this promoter region mutation and several coding region mutations of the TIGR/MYOC gene. The presence of mutations in the coding region has been associated with an increased probability of developing the disease.[5] This is a noninvasive in-office test in which individual DNA sample is collected using cheek brushes. Some patients appear predisposed to development of aggressive form of the disease. OcuGene offers the clinician the mean to identify people at risk, particularly genetically predisposed nonglaucomatous family members who cannot be diagnosed with current glaucoma testing. The test is positive in 15–20% of POAG patients and 99% sensitive. It is useful for both diagnostic and prognostic purposes. Negative test is reassuring for both the patient and the doctor, whereas positive test indicates aggressive disease and necessitates treatment and closer monitoring of the patient (internal data, InSite Vision).[5]

## Methods of Genetic Testing

1. Direct sequencing which is quite time consuming and expensive automated DNA sequencing that consists of polymerase chain reaction (PCR) amplification, sequence-specific fluorescent labeling, and liquid chromatography are some of the available methods of genetic testing. Direct sequencing is perhaps the most robust single method for mutation detection today. It can evaluate more than 600 contiguous nucleotides in the genome in both directions for about $12.
2. *Screening methods* include single-strand conformational polymorphism analysis (SSCP) and denaturing high performance liquid chromatography (DHPLC) when multiple samples are to be examined. SSCP can detect the common *CEP290* variation in Leber congenital amaurosis for not more than $0.50 per person and is so simple to perform that thousands of samples can be analyzed in a single day by a single laboratory.[6,7]
3. *A multiplatform approach*[2]: The much less sophisticated SSCP can be combined with automated DNA sequencing to dramatically decrease the cost and increase the speed of a genetic test in a specific clinical situation. If DNA sequencing alone was employed, this same work might take three times longer and would cost more than six times.[6,7]

## Who Could Potentially Benefit from Genetic Testing for glaucoma?

At present, glaucomatous optic neuropathy can be diagnosed only when damage in the form of either optic nerve axonal loss or visual function deficits is already present. The use of genetic tools makes possible both early diagnosis, before any irreversible damage has occurred, and early treatment. In addition, ruling out the existence of glaucoma can eliminate unnecessary treatment, lifelong follow-up, and perhaps, just as important, lifelong anxiety with its effect on the individual’s quality of life. Genetic testing may be particularly relevant to the following groups, who pose difficult diagnostic and therapeutic problems:[8]

1. *Ocular hypertensive* subjects who have repeated measurements of increased IOP, but lack established glaucoma damage.
2. *Glaucoma suspects*, including subjects with suspicious-looking discs and those with stable (or atypical) visual field abnormalities. At present, it is unclear for which of these glaucoma suspects treatment is indicated.
3. *Patients with early glaucoma* in which the question arises whether to use aggressive therapy; such as initial trabeculectomy, or rather to start the step-wise approach with a single medication. Genetic testing may assist in tailoring treatment to an individual’s long-term prognosis. Because prognosis assessment is extremely vague, target IOPs are based, at present, only on documented deterioration. In the future, genetic data could be used for determining target IOPs, on the basis of future prognosis rather than past deterioration.
4. Individuals who are at an increased risk for glaucoma due to a *positive family history, or other risk factors*, such as pseudoexfoliation or pigmentary dispersion syndromes.

## How Could Genetic Input Benefit Clinical Management of Glaucoma?

Current therapeutic options for ameliorating glaucoma involve lowering of IOP. Although neuroprotective drugs as therapy for glaucoma are promising, the use of this potential treatment option could be enhanced by early diagnosis. Patients already diagnosed with glaucoma could benefit from genetic screening if the test results could predict the severity and rapidity of disease progression. Treatment options range from mild (a β-blocker) to aggressive (initial trabeculectomy), predicting future disease behavior could help tailor therapy to the needs of an individual.

## Target Tissue, Mode of Delivery of Gene Therapy and Potential Predicted Effects

Gene therapy of ocular disorders benefits from the accessibility of the eye, the ability to visualize the diseased tissue, and the large number of specific gene defects known to be responsible for many inherited eye disorders.[9] Mutations in the DNA sequence of a particular gene can result in a protein product that is *not produced, works poorly, or has adopted a novel function that is detrimental to the cell*.[10]

Gene therapy is a technique for correcting defective genes responsible for disease development. Theoretically, a normal copy of the gene can physically take the place of the flawed gene and restore the gene function of the cell. In practice, however, actually replacing the flawed gene with a normal gene is a difficult task.[11] Currently, the aim of gene therapy is to add a useful gene to the cell or tissue that suffers the consequences of the flawed gene. In some cases, the new gene may code for an entirely different protein whose function compensates for the protein encoded by the flawed gene.[12]

In general, most of the current approaches to gene therapy are aimed at repairing the somatic cells of the particular tissue affected by the disease gene.[13] Gene delivery may be tailored to the somatic cells affected by the disorder. Specific treatment of the diseased cells does not affect the other cells of the body, which include the germline cells. Because the germline cells continue to carry flawed copies of the gene, the disease may still be passed to offspring of the affected patient. Gene therapy targeted to germline cells as well as the diseased somatic cells results in successful treatment of the disease in the affected individual and prevents transfer of the disease to any offspring.[14]

A general approach to gene therapy is to use an altered (recombinant) virus to carry the gene of interest to the desired tissue. Using genetic engineering techniques, the viral DNA is modified so that the viral genes required for virus proliferation are removed and the therapeutic gene is put in their place. Such a virus may invade the diseased tissue, become incorporated into the host DNA, and express the desired gene. Because the modified virus does not have the viral genes required for viral replication, the virus cannot proliferate, and the host cell does not die.[15]

Diseases caused by mutations that create a gene product destructive to the cell need to be treated using a different approach. In these cases, genes or oligonucleotides that may inactivate the mutated gene are introduced into the cell. This is called “*antisense therapy*,” and it is proving to be a useful approach for diseases caused by the “gain of function mutations.” A number of different viral vectors likely to be useful for gene therapy are currently under investigation. In addition, evaluation of nonviral mechanisms for the introduction of therapeutic genes into diseased tissue is ongoing.

There are several approaches for correcting faulty genes:

1. Inserting a normal gene into a nonspecific location within the genome to replace a nonfunctional gene.
2. An abnormal gene could be swapped for a normal gene through homologous recombination; this approach is the most common.
3. The abnormal gene could be repaired through selective reverse mutation, which returns the gene to its normal function.
4. The regulation (the degree to which a gene is turned on or off) of a particular gene could be altered.

## Gene Transfer Techniques

**Figure 1:**
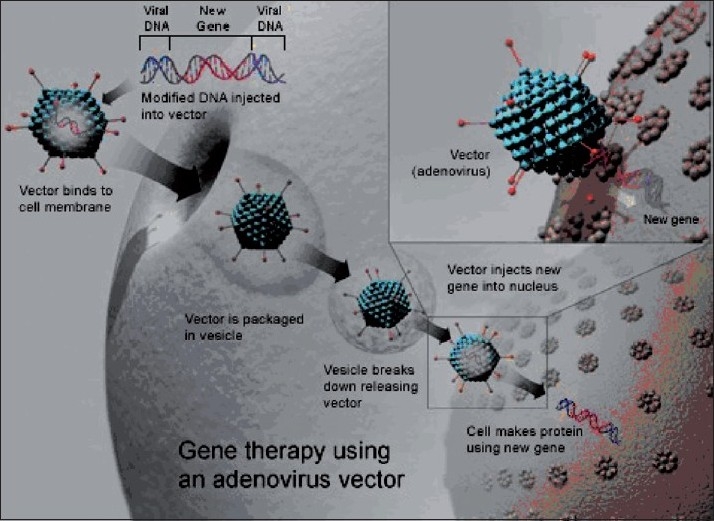
Modified virus vector in which a new gene is incorporated and delivered to the diseased cell

Gene therapy works by delivering the therapeutic gene to the patients’ target cells through the carrier molecule called a vector. Currently, the most common vector is a virus that has been genetically altered to carry normal human DNA. Once the vector enters target cells, it unloads its genetic material containing the therapeutic human gene. The generation of a functional protein product from the therapeutic gene restores normal cell function [Figure 1].

Four basic prerequisites should be met for any genetic therapy targeted to an ocular disease: (1) An efficient and nontoxic gene delivery technique. (2) Sufficient characterization of the genetic basis of the disease to select an appropriately matched therapeutic approach. (3) Proper control of the expression of the therapeutic gene. (4) The availability of an animal model of the disease for preclinical testing. Glaucoma is a disease in which some of these conditions can be met. Strategies now exist that use gene therapy to modulate aqueous production and outflow and prevent RGC death.[16,17]

The clinical application of gene transfer can be accomplished in either of two ways: *in vivo* or *ex vivo*. During *in vivo* gene transfer, the foreign gene is injected into the patient by viral and nonviral methods. The *in vivo* gene delivery systems are summarized in Table 2. In contrast, an ex vivo gene transfer involves a foreign gene transduced into the cells of a tissue biopsy, outside the body, and then resulting genetically modified cells are transplanted back into patient.

**Table 2:** Summary of *in vivo* gene delivery systems

*Viral Methods*		*Non Viral Methods*	
*Viruses serving as viral vectors*	
Retroviruses Adenoviruses Adeno-associated viruses	Micro Seeding Gene Therapy:	Direct injection of therapeutic DNA into target cells using a gene gun. This approach is limited in its application because it can be used only with certain tissues and requires large amounts of DNA (simplest method).
Herpes simplex viruses Lenti virus	Cationic Liposomes:	Creation of artificial lipid spheres with an aqueous core. This liposome, which carries the therapeutic DNA, is capable of passing the DNA through the target cell’s membrane.
	Macromolecular Conjugate:	Here therapeutic DNA gets inside target cells by chemically linking the DNA to a molecule that will bind to special cell receptors. Once bound to these receptors, the therapeutic DNA constructs are engulfed by the cell membrane and passed into the interior of the target cell. This delivery system tends to be less effective than other options.
	Gene Activated Matrices (GAMS):	These deliver naked DNA via polymer matrix sponges.

## Gene Delivery Systems and Target Tissues

Because of their role in aqueous production, drainage, and pathogenesis of glaucomatous damage, these are appropriate target structures or cell types for glaucoma gene therapy: trabecular meshwork (TM), ciliary epithelium (CE), ciliary muscle (CM), RGCs, and Muller cells (MCs). To date, six delivery systems have been tested for ability to deliver genes to the relevant tissues or cells. These are summarized in Table 3 and include adenoviruses (Ads), adeno associated viruses (AAVs), herpes simplex viruses (HSVs), lentiviruses (LVs; Feline Immunodeficiency Virus [FIV] and Human Immunodeficiency Virus [HIV]), Liposomes (LPs), and Naked DNA.[18]

**Table 3:** Potential target genes and tissues that could be used therapeutically to treat glaucoma[19]

*Tissue type*	*Target gene*	*Predicted effect*
Trabecular Meshwork	Cytoskeleton regulatory proteins	Disruption of cellular cytoskeleton stimulates an increase in aqueous outflow
	Myocilin	High-expressing wild-type allele to compete mutant allele
	Metalloproteinases	Extracellular matrix remodeling
Ciliary Epithelium	Genes that regulate circadian rhythm of aqueous production	Reduce nighttime increases in aqueous production that lead to potentially damaging IOP levels
	B-Adrenergic receptors	Enhance potential of ciliary body cells to respond to drugs that inhibit aqueous production
	Other genes modulating fluid production	Modulate TM and CM functions
	Neuropeptides	
Ciliary Muscle cells	Gene X	Upregulation of prostaglandin synthesis
	Metalloproteinases	Produce matrix metalloproteases to enhance uveoscleral outflow
Retinal Ganglion Cells	Neurotrophin receptors (TrkB) Neurotrophin genes	Increase the potential for RGCs to respond to neurotrophins
	BcIX	Enhance levels of endogenous antiapoptosis gene product antagonize BAX function
	Bax	Antisense construct to reduce levels of BAX protein
	Hsp70/72	Enhance the endogenous stress response of RGCs to resist damaging stimuli
Muller ells	GLAST	Upregulate the endogenous glutamate transporter to enhance clearance of extracellular glutamate levels
	Neurotrophins	Provide a surrogate source of neurotrophins for RGCs

### A. Anterior segment delivery systems

Several studies have established that adenovirus vectors (Ad vectors) can deliver transgenes very efficiently to the TM after intracameral injection. Because of the natural flow of aqueous humor, intracameral delivery of vectors carries the viruses directly to the TM. Recombinant Ads transduce all TM cell types with high efficiency in all species so far investigated (rabbits, mice, rats, dogs, monkeys, and anterior segments from postmortem human donors). Adeno associated virus (AAV) vectors appear to be unsuitable for anterior segment delivery.[19–21]

HSV vectors are capable of efficient gene delivery to structures and cells relevant to glaucoma. In monkeys and rodents, intracameral injection results in efficient delivery to cells in the TM and CE.[22,23] Only the CMV promoter has been tested with HSV vectors, and gene expression has lasted only 10 days, at most. Vector DNA persists at least 1 month after transduction, suggesting that a promoter shutoff is involved.

Delivery with LV vectors based on HIV and FIV has also been tested. Efficient delivery occurs in the TM with both types of vectors.[24] One potential advantage of LV vectors is the ability to integrate into the host cell genome of nondividing or slowly dividing TM cells, which may result in increased duration of expression. The major drawback to integrating vectors is the potential for insertional mutagenesis, which although rare, is a serious concern.

Liposome-mediated plasmid delivery has also been achieved. TM cells are transduced, but the efficiency is low.[25] Toxicity due to certain types of liposomes or specific constituents has also been seen. Transfer of naked DNA to anterior chamber structures has not been reported to date. Successful delivery of naked DNA to cells at the time of glaucoma filtration bleb surgery has also been achieved; efficient gene delivery to the CM *in vivo* has not been reported to date.

### B. Posterior segment delivery systems

It is now well established that intravitreal delivery is the preferred route to deliver genes to the RGCs. Intravitreal injection of Ad vectors results in efficient delivery to MCs. For reasons not yet clear, Ad gene transfer to the RGCs is very limited.[26] MCs, however, appear to be important sources of neurotrophic factors, and delivery of such secreted factors or modulation of their expression in these cells could be important in neuroprotective strategies to protect RGCs. AAV appears to have selective, stereotype-specific tropism for the RGCs.

Intravitreal injections in the rat result in up to a 72% transduction efficiency, with optimal expression occurring between 2 and 4 weeks after injection in rodents.[27] This high efficiency may be due to the expression of membrane associated heparin sulfate proteoglycans in RGCs, which mediate attachment of AAV to cells. Heparin sulfate proteoglycans are also receptors for HSV.[28] Delivery of HSV results in efficient transduction of RGCs. Efficiencies of up to 50% can be achieved with a single intravitreal injection in rats.[22] Table 4 shows the glaucoma relevant tissues and vector system available.

**Table 4:** Glaucoma relevant tissues and available vector systems[19]

*Tissue Type*	*Vector*	*Route*
Trabecular Meshwork	Adenovirus	Intracameral
		Intracameral
	Adeno-associated virus serotypes 2, 3, 4	Intracameral
		Tissue culture
	Herpes simplex virus	Intracameral
	Lentivirus	Intracameral
	Liposomes	Intracameral
Ciliary Epithelium	Adenovirus	Intracameral
		Lens culture
	Adeno-associated virus	
	Herpes simplex virus	Intracameral
	Lentivirus	
	Liposomes	
Ciliary Muscle	Adenovirus	
	Adeno-associated virus	
	Herpes simplex virus	Tissue culture
	Lentivirus	
	Liposomes	
Retinal Ganglion	Adenovirus	Intravitreal
Cells		
	Adeno-associated virus	Intravitreal
	Herpes simplex virus	Intravitreal
		Retrograde
	Lentivirus	
	Liposomes	

## Toxicity

Direct toxicity to transduced cells does not appear to be a problem with the vectors tested to date. The most common negative response has been the induction of an inflammatory response composed predominantly of monocytic cellular infiltrates in the anterior chamber.[18] The most likely explanation for the effect is the induction of proinflammatory cytokines by the vector viruses. Vehicle components and injection *per se* do not appear to be involved, because inflammation is not seen in control eyes. The induction of inflammation is dose dependent, because reducing the amount of vector with Ad and HSV vectors eliminates inflammation.[20] However, this reduces transduction efficiency, particularly with HSV vectors. Both AAV and LV vectors do not appear to induce inflammation, but most of these studies have been performed in rodents, and it is clear that species differences also play a role.[18]

## Potential Target Genes for Relevant Tissues and Predicted Effects

Although the genetic basis of most glaucomas remains unknown, the transfer, modulation, and expression of genes encoding IOP lowering and/or neuroprotective gene products may serve to modify the physiology of relevant cells and block the pathogenesis of the disease. As with other chronic diseases, the use of genes to treat glaucoma will provide improved efficiency and a longer duration of effect. Table 3 shows the potential target genes and tissues that could be used therapeutically to treat glaucoma.[18]

Lowering IOP by manipulating the tissues of the anterior segment with gene therapy could represent the first immediate treatment of glaucoma. The TM, CE, and CM are all potential targets. The TM’s juxtacanalicular region and inner wall of Schlemm’s canal constitute the primary barrier to aqueous humor before it leaves the eye. Manipulation of the biochemistry of the cells and extracellular matrix in these regions has the potential to modulate outflow resistance and lower IOP. Investigators have successfully transferred genes to this tissue with different vectors and through different routes of administration [Table 4].[18]

Several investigators deliver potential physiologically relevant genes, in addition to reporter genes. Kee *et al*.[21] have demonstrated that an Ad vector carrying the metalloproteinase stromelysin can be transduced to TM cells of rats after intracameral injection. In human postmortem perfused organ cultures, Ad vectors carrying wild-type myocilin and genes that affect the cytoskeleton increase outflow facility.[29,30] Lentivirus vector was also used with high efficiency to transduce the human trabecular meshwork (HTM) with overexpression of metalloproteinases, green fluorescent proteins (GFP) for at least 10 months after injection.[31] In addition, AAV had been used by Borras *et al*. and demonstrated genes and mechanisms which lead for the first time to efficient AAV transduction of the HTM.[32]

The final common outcome of glaucoma is RGC death. The stimulatory mechanism or mechanisms causing RGC death are unclear, but several strategies to block RGC apoptosis are available. The goal of gene therapy in glaucoma, therefore, is to slow the rate of RGC death. There are a number of strands of evidence that suggest brain-derivied neurotrophic factor (BDNF) as a potential neuroprotective agent in glaucoma. RGC are trophically dependent on BDNF, retrogradely transported from target areas in the brain to ganglion cell bodies in the retina.

Several investigators are now exploring the use of gene transfer to deliver a neurotrophic factors. Ad-mediated intravitreal delivery of BDNF has been shown to protect RGCs in a rat optic nerve transection model.[26] MCs are transduced by Ad and enhancement of RGC survival is due to secretion of BDNF from these cells. Even though the expression of the transgene was prolonged to 1 month by the use of immunosuppressants, the protective effect was transient.[27] Furthermore, AAV-mediated TrkB gene transfer into RGCs combined with exogenous BDNF administration markedly increases neuronal survival.[27]

In addition, AAV incorporating cDNA was used for BDNF to transfect RGC in a rat model of glaucoma. A high proportion of RGC were transfected after a single intravitreal injection. After 4 weeks of experimental glaucoma, eyes that received intravitreal AAV-BDNF 38% of RGC were rescued by the AAV-BDNF.[33,34] Other studies have shown that AAV can mediate transgene expression in RGC for at least 1 year.[35] Isenmann *et al*.[36] also found protection of the RGCs after Ad-mediated delivery of BDNF, and protection was increased by the combined systemic administration of the free radical scavenger *N-tert*-butyl-(2-sulfophenyl)-nitrone (S-PBN). Similar RGC survival results were obtained recently with Ads containing the ciliary neurotrophic factor (CNTF).[37]

Another approach to gene therapy in glaucoma has recently been reported by McKinnon and coworkers. They have taken a different approach, involving the modulation of pathways responsible for apoptosis in RGC. The final common pathway of RGC apoptosis involves the activation of caspase enzymes that participate in the mechanisms of cell suicide. McKinnon *et al*.[38] injected an AAV vector coding for human baculoviral IAP repeat-containing protein-4 (BIRC4), a potent caspase inhibitor, into one eye of rats. BIRC4 promoted RGC survival, presumably by inhibiting the enzymatic completion of apoptosis.[38,39]

Gene therapy could also be used to *prevent the proliferative wound-healing response* that follows glaucoma filtration surgery. Using an Ad vector encoding the cell cycle inhibitor p21 (rAd-p21). Perkins *et al*.[40] inhibited proliferation of Tenon’s fibroblasts in rabbits with a single administration of rAd-p21 (by applying a vector-soaked sponge for 5 min). This resulted in the maintenance of functional filtration blebs at 30 days after surgery without the severe tissue effects observed in the mitomycin-treated eyes. Other investigators have used naked DNA to transfer the reporter gene chloramphenicol acetyltransferase to the same cells.[41]

Antiproliferative gene therapy has been used adjunctively with glaucoma surgery in animal models. A recombinant adenovirus (rAd) was used to introduce the human gene for p21^WAF-1/cip-1^ (p21) into rabbit Tenon’s fibroblasts in a normotensive model of glaucoma surgery.[42] This transgene product normally blocks entry of dividing cells into S phase of the cell cycle by antagonizing the activity of several cyclin-dependent kinases. The expression of p21 in the rabbit model persisted for more than 2 weeks after transduction and was as effective as mitomycin C (MMC) at reducing IOP and preventing fibroproliferation over a 4-week period. Unlike MMC, however, the p21 gene therapy produced no toxic effects in rabbits.[43]

Gene therapy using naked plasmid DNA and a simple collagen shield delivery vehicle or injected directly subconjunctivally may be useful for regulating wound healing after glaucoma surgery. Transfection of filtration tissues is enhanced by absorption of naked DNA into a collagen shield soaked in a solution containing the plasmid. Furthermore, transfection is localized to the fibroblasts and inflammatory cells of the filtration bleb site.[41]

## Small Interfering RNA (siRNA)

An interesting poster presented in the ARVO meeting by Jimenez *et al*. 44about the therapeutic use of siRNA (small interfering RNA) in OHT and glaucoma treatment. siRNA holds great therapeutic promise for gene silencing in a nontoxic and highly effective way, as it is naturally used by cells to regulate gene expression. In this study, the effect of siRNAs targeting different isoforms of ATPases and cyclooxygenases on IOP was investigated in rabbits. Latanoprost and dorzolamide were used as control drugs. The results showed that IOP decreases by siRNA were comparable with those by commercially available drugs. The IOP-lowering effect of siRNA lasted longer (about 100 h) than that of commercially available drugs.

## Limitations of Gene Therapy

The following are limiting factors on the use of gene therapy:

1. Short-lived nature of gene therapy.
2. Immune response of the patient.
3. Problems with viral vectors such as patient-toxicity, immune and inflammatory responses, and gene control and targeting issues.
4. Limitation of sufficient quantity of the engineered gene that can be delivered.
5. Extreme cost.
6. Ethical restrictions.

Despite the above limitations, the future hold promises for gene therapy in glaucoma. Gene therapy could be used in two ways in glaucoma: as a drug delivery system, and as a basis for developing new therapies and treatment end points based on the genetic mutations that cause glaucoma.[4] If indeed the TIGR/MYOC gene or separate genes could be shown to be a risk factor for earlier onset or more progressive disease, a patient’s therapeutic end points could be modified based on his or her genetic profile. In the future, such a patient may start therapy earlier in life and be maintained at a lower IOP to help prevent glaucomatous progression and visual loss. Consequently, the genetic profile may help individualize patient therapy to better ensure a stable glaucomatous disease course.
